# Characterization of antibiotic and disinfectant susceptibility in biofilm-forming *Acinetobacter baumannii*: A focus on environmental isolates

**DOI:** 10.1556/1886.2024.00014

**Published:** 2024-03-05

**Authors:** Péter Pallós, Márió Gajdács, Edit Urbán, Yvett Szabados, Klaudia Szalai, Lívia Hevesi, Anna Horváth, Anna Kuklis, Devina Morjaria, Wajiha Iffat, Helal F. Hetta, Nicola Piredda, Matthew Gavino Donadu

**Affiliations:** 1 Department of Oral Biology and Experimental Dental Research, Faculty of Dentistry, University of Szeged, Tisza Lajos krt. 64-66., 6720 Szeged, Hungary; 2 Department of Medical Microbiology and Immunology, University of Pécs Medical School, Szigeti út 12, 7624 Pécs, Hungary; 3 Department of Pharmaceutics, Dow College of Pharmacy, Faculty of Pharmacy and Pharmaceutical Sciences, Dow University of Health Sciences, OJHA Campus, Karachi, Pakistan; 4 Department of Natural Products and Alternative Medicine, Division of Microbiology and Immunology, Faculty of Pharmacy, University of Tabuk, Tabuk 71491, Saudi Arabia; 5 Department of Medical Microbiology and Immunology, Faculty of Medicine, Assiut University, Assiut, 71515, Egypt; 6 Radiology Unit, Giovanni Paolo II Hospital, ASL Gallura, 07026 Olbia, Italy; 7 Hospital Pharmacy, Giovanni Paolo II Hospital, ASL Gallura, 07026 Olbia, Italy; 8 Department of Medicine, Surgery and Pharmacy, Scuola di Specializzazione in Farmacia Ospedaliera, University of Sassari, 07100 Sassari, Italy

**Keywords:** Acinetobacter baumannii, antimicrobial resistance, antiseptic resistance, MDR, biofilm-formation, environmental, phenotypic assay

## Abstract

The clinical role of *Acinetobacter baumannii* has been highlighted in numerous infectious syndromes with a high mortality rate, due to the high prevalence of multidrug-resistant (MDR) isolates. The treatment and eradication of this pathogen is hindered by biofilm-formation, providing protection from noxious environmental factors and antimicrobials. The aim of this study was to assess the antibiotic susceptibility, antiseptic susceptibility and biofilm-forming capacity using phenotypic methods in environmental *A. baumannii* isolates. One hundred and fourteen (*n* = 114) isolates were collected, originating from various environmental sources and geographical regions. Antimicrobial susceptibility testing was carried out using the disk diffusion method, while antiseptic susceptibility was performed using the agar dilution method. Determination of biofilm-forming capacity was carried out using a microtiter-plate based method. Resistance in environmental *A. baumannii* isolates were highest for ciprofloxacin (64.03%, *n* = 73), levofloxacin (62.18%, *n* = 71) and trimethoprim-sulfamethoxazole (61.40%, *n* = 70), while lowest for colistin (1.75%, *n* = 2). Efflux pump overexpression was seen in 48.25% of isolates (*n* = 55), 49.12% (*n* = 56) were classified as MDR. 6.14% (*n* = 7), 9.65% (*n* = 11), 24.65% (*n* = 28) and 59.65% (*n* = 68) of isolates were non-biofilm producers, weak, medium, and strong biofilm producers, respectively. No significant differences were observed between non-MDR vs. MDR isolates regarding their distribution of biofilm-producers (*P* = 0.655). The MIC ranges for the tested antiseptics were as follows: benzalkonium chloride 16–128 μg mL^−1^, chlorhexidine digluconate 4–128 μg mL^−1^, formaldehyde 64–256 μg mL^−1^ and triclosan 2–16 μg mL^−1^, respectively. The conscientious use of antiseptics, together with periodic surveillance, is essential to curb the spread of these bacteria, and to maintain current infection prevention capabilities.

## Introduction

Despite the systemic implementation of infection prevention and control (IPC) strategies, non-fermenting Gram-negative bacteria (NFGNB) remain as common colonizers an as etiological agents of healthcare-associated infections (HAIs) in patients affected by immunosuppression, invasive medical interventions or other chronic conditions [1, 2]. Among NFGNB, the *Acinetobacter baumannii-calcoaceticus* complex contains some of the most frequently isolated species from clinical specimens, but they are also commonly found in environmental sources, as these bacteria may survive for several months on abiotic surfaces [3, 4]. The clinical role of *A. baumannii* has been previously described in ventilator-associated pneumonia (VAP), bacteremia, urinary tract infections, exposure keratitis and wound infections, among others [5, 6]. According to recent reports, invasive *A. baumannii* infections are associated with considerable overall mortality rates, both in the case of community-acquired infections (0–64%) and for HAIs (23–68%) [7]. Additionally, the meta-analysis of Lim et al. – involving 114 studies – reported an overall mortality rate for *A. baumannii* VAP at 42.6%, however, this may be as high as 84.0% in patients treated in intensive care units (ICUs) [8].

Antimicrobial resistance (AMR) has become one of the major issues for global health, as the available number of antibiotics left, useful for the treatment of difficult-to-treat infections is alarmingly low [9]. *A. baumannii* possesses a combination of intrinsic resistance resistance mechanisms, in addition to the remarkable ability of this pathogen to acquire resistance determinants [10, 11]. Multidrug-resistant (MDR) and extensively drug-resistant (XDR) strains of *A. baumannii* are some of the most concerning pathogens in clinical practice [12]. According to the European Centre for Disease Prevention and Control “Surveillance Atlas of Infectious Diseases”, resistance rates of *Acinetobacter* spp. for aminoglycosides, fluoroquinolones and carbapenems in Hungary were 68.8, 78.1 and 48.1% in 2012, while 47.2, 63.8 and 57.9% in 2022, respectively [13]. Furthermore, carbapenem-resistant *A. baumannii* was designated by the World Health Organization (WHO) as a “Priority 1: Critical” pathogen, on their “Priority Pathogens List”, for which novel antimicrobials are desperately needed [14]. In addition to antibiotics, effective healthcare heavily relies on the availability of effective antiseptics and disinfectants (e.g., benzalkonium chloride, chlorhexidine digluconate, ethyl-alcohol, formaldehyde, hydrogen-peroxide, povidone iodine, quaternary ammonium compounds (QACs), triclosan) [15]; these compounds are essential for antisepsis, hand hygiene, and the elimination of bacterial reservoirs [16]. While many epidemiological studies report on the resistance rates of these pathogens globally, the data on rising rates of antiseptic and disinfectant resistance is scarce, however, these resistance mechanisms should be appreciated during IPC initiatives [17]. For example, it was shown that hands heavily contaminated (10^6^ colony-forming units [CFU]/fingertip) with *A. baumannii* could survive the effects of many antiseptics and disinfectants, or that these bacteria could survive in soap dispensers [18, 19].

For *Acinetobacter* spp., biofilm-formation is one of the main virulence factors *in vivo*, providing protection against the immune system and antimicrobials, and furthermore, it also allows for survival in harsh environmental conditions [20]. Therefore, biofilm-formation is often termed as a tertiary form of “adaptive” resistance against antimicrobial drugs [21]. Since the availability of laboratory methods to study the biofilm-forming capacity of bacterial isolates, there has been substantial interest in assessing whether co-regulation exists between biofilm formation, the expression of resistance genes and the MDR phenotype [22, 23]. In a previous study, we aimed to assess the possible relationship between biofilm-formation and antibiotic resistance in a large selection of clinical *A. baumannii* isolates [24]; our experiments showed no significant associations between the MDR status of the isolates and biofilm-forming capacity. To corroborate and confirm our previous results, the aim of the present study was to investigate the relationship between biofilm-forming capacity and antibiotic resistance in *A. baumannii* isolates originating from various environmental sources and geographical regions, in addition, to study the disinfectant susceptibility levels of biofilm-forming isolates.

## Materials and methods

### Sample size determination

The initial sample size required from environmental *A. baumannii* isolates was determined using formula (1) shown below, based on the recommendations of Thrusfield et al. [25], where *n* was the calculated sample size, *z* was the desired confidence level (1.96), *i* was the standard sampling error (5%), while *p* was the estimated prevalence set at 5% [26]. Based on the calculation, the required sample size of *n* = 114 isolates was determined.

(1) $$n=\frac{z^{2}p\left(1-p\right)}{i^{2}}$$

### Collection of isolates

A total of one hundred and fourteen (*n* = 114) isolates were included in the study, which were obtained from strain collections of various geographical regions and environmental origins (i.e. Karachi [Pakistan], Olbia [Italy] and Szeged [Hungary]), sourced from areas of high rates of anthropogenic presence. Environmental sampling procedures were performed based on previously described protocols [27]. As a general rule, only one *A. baumannii* isolate per source was included [24]. During the experiments, *A. baumannii* clinical isolate no. 59738 (a MDR isolate, weak biofilm producer) and *A. baumannii* ATCC 19606 (susceptible isolate, strong biofilm producer) were used as control strains (the latter was obtained from the American Type Culture Collection, Manassas, VI, USA) [28]. Stock cultures were stored at −80 °C in a cryopreservation medium (700 µL trypticase soy broth + 300 µL 50% glycerol) until further use.

### Identification of *A. baumannii* isolates

Before further analysis, *A. baumannii* isolates were re-identified by matrix-assisted laser desorption/ionization–time-of-flight mass spectrometry (MALDI–TOF MS; MicroFlex MALDI Biotyper, Bruker Daltonics, Bremen, Germany), according to methodologies previously described [24]. Reliable species-level identification was accepted in the case of a log(score) value ≥ 2.30 [29].

### Antimicrobial susceptibility testing

Antimicrobial susceptibility testing (AST) for *A. baumannii* isolates was carried out according to the standard disk diffusion method (Oxoid, Basingstoke, UK) on Mueller-Hinton agar plates (bioMérieux, Marcy-l’Étoile, France), during which, the following antibiotics were tested: aminoglycosides (gentamicin [10 µg disk], amikacin [30 µg disk]), carbapenems (imipenem [10 µg disk], meropenem [10 µg disk]), fluoroquinolones (ciprofloxacin [5 µg disk], levofloxacin [5 µg disk]), trimethoprim-sulfamethoxazole [23.75/1.25 µg disk] and colistin [10 µg disk]. With the exception of colistin, interpretation of the results was carried out according to the standards and breakpoints of the European Committee on Antimicrobial Susceptibility Testing (EUCAST) v. 11.0 [30]. Results indicating “susceptible, increased exposure (I)” were grouped with and reported as susceptible (S) [31]. Susceptibility to colistin was assessed according to the provisional breakpoints, as advised by Galani et al. [32]. Classification of the isolates as MDR (i.e. resistant to at least one agent in ≥3 antibiotic groups) was based on the recommendations of Magiorakos et al. [33].

### Phenotypic detection of efflux pump overexpression

The overexpression of resistance-nodulation-division-type (RND) efflux pumps was assessed if ciprofloxacin-resistance was noted based on the disk diffusion test, described previously. The assay was carried out using a phenylalanine-arginine β-naphthylamide (PAβN)-based agar dilution method, as recommended by Khalili et al. [34]. An isolate was considered positive for efflux pump overexpression, if a two-fold decrease in ciprofloxacin minimum inhibitory concentrations (MICs) was noted by E-tests (Liofilchem, Roseto degli Abruzzi, Italy) in the presence of PAβN, compared to the MIC values without the inhibitor [35].

### Biofilm-formation assay

Determination of biofilm-forming capacity of environmental *A. baumannii* was carried out using a microtiter-plate based method, as previously described by Ramos-Vivas et al. [36]. Briefly, overnight *A. baumannii* cultures, grown on Luria–Bertani (LB) agar, were inoculated into 5 mL of LB-broth and incubated overnight at 37 °C. The next day, a 20 μL of bacterial suspension (set at 10^6^ CFU mL^−1^ density) and 180 μL of LB-broth were transferred onto 96-well flat-bottomed microtiter plates to a final volume of 200 µL. Following a 24 h incubation period at 37 °C, supernatants were discarded, and the wells were washed three times using 200 µL of phosphate-buffered saline (pH set at 7.2) to remove planktonic cells. The wells were then fixed with 250 μL of methanol (Sigma-Aldrich, St. Louis, MO, USA) for 10 min, and stained with a 1.0% crystal violet solution for 15 min (CV; Sigma-Aldrich, St. Louis, MO, USA). The CV dye was then discarded, and the wells were washed three times with purified water to remove excess stain. The wells' contents were solubilized in 250 μL of 33% v/v% glacial acetic acid (Sigma-Aldrich, St. Louis, MO, USA), and a microtiter plate reader was used to measure and record absorbance values at 570 nm (OD_570_) as mean ± standard deviation (SD). Isolates were then classified according to their biofilm-forming capacity, based on the recommendations of Stepanovic et al. [37]; a cut-off value of optical density (OD_c_) was calculated using the following formula: OD_c_ = average OD of the negative control + (3 × standard deviations of negative control). Subsequently, isolates were classified into the following categories, based on their OD_570_ measurements: strong biofilm producer (OD > 4 × OD_c_); medium biofilm producer (4 × OD_c_ ≥ OD > 2 × OD_c_); weak biofilm producer (2 × OD_c_ ≥ OD > OD_c_); and non-biofilm producer (OD ≤ OD_c_), respectively [37].

### Disinfectant susceptibility testing

The MICs of antimicrobial disinfectant agents in medium and strong biofilm-producers were determined according to the agar dilution method, based on the Clinical and Laboratory Standards Institute (CLSI) recommendations. The following disinfectants were tested in our experiments: benzalkonium chloride (95%), chlorhexidine digluconate (20%), formaldehyde (38%) and triclosan (98%) (all purchased from Sigma-Aldrich, St. Louis, MO, USA). Serial 2-fold dilutions of disinfectants were prepared in Mueller-Hinton agar (bioMérieux, Marcy-l’Étoile, France) in the concentration range between 0.125 and 1,024 μg mL^−1^ [38]; then, 1–2 μL of bacterial suspension was spotted on the agar surfaces. Incubation of the plates was carried out in a 37 °C air thermostat for 18–24h. The MIC was recorded as the lowest concentration of disinfectant to inhibit the growth of the organisms [38]. MIC_50_ and MIC_90_ were defined as the as the lowest concentrations of compounds to inhibit the growth of 50 and 90% of isolates, respectively.

### Statistical analysis

All continuous variables were expressed as means and standard deviations (mean ± SD), whereas categorical variables were expressed as frequencies (n) and percentages (%). The Fisher-exact test was used to detect associations between biofilm-forming capacity and MDR-status (with Cramér's phi [φ] effect size measure). Statistical analysis were performed using SPSS software version 22.0 (IBM Corp., Armonk, NY, USA). *P* < 0.05 was considered statistically significant.

### Ethical considerations

The study was conducted in accordance with the Declaration of Helsinki and national and institutional ethical standards. Ethical approval for the study protocol was obtained from the Human Institutional and Regional Biomedical Research Ethics Committee, University of Szeged (registration number: 140/2021-SZTE [5019]).

## Results

### Antimicrobial resistance and efflux pump overexpression in environmental *A. baumannii*

Resistance rates of environmental *A. baumannii* included in our study was as follows (in decreasing order): ciprofloxacin 64.03% (*n* = 73), levofloxacin 62.18% (*n* = 71), trimethoprim-sulfamethoxazole 61.40% (*n* = 70), imipenem 56.14% (*n* = 64), meropenem 56.14% (*n* = 64), gentamicin 42.11% (*n* = 48), amikacin 31.57% (*n* = 36) and colistin 1.75% (*n* = 2). Out of these isolates 49.12% (*n* = 56) met the criteria to be classified as MDR. Overexpression of RND-type efflux pumps was assessed in ciprofloxacin-resistant isolates, using a plate-based assay: 75.34% (*n* = 55 out of 73 isolates; 48.25% overall) of isolates were positive.

### Biofilm-forming capacity in environmental *A. baumannii*

The measurement of biofilm-formation in environmental *A. baumannii* was carried out in 96-well microtiter plates, on the basis of CV staining and spectrophotometric measurement. To calculate classification breakpoints, the OD_570_ values for the negative control (clinical isolate no. 59738) and the positive control (ATCC 19606) were also measured, which corresponded to 0.088 ± 0.016 and 0.507 ± 0.092, respectively. Thus, the following breakpoints were set during our analyses: OD_c_ = 0.136, non-biofilm producer: OD ≤ 0.136, weak biofilm producer: 0.272 ≥ OD > 0.136, medium biofilm producer: 0.544 ≥ OD > 0.272, and strong biofilm producer: OD > 0.544. Accordingly, 6.14% (*n* = 7), 9.65% (*n* = 11), 24.65% (*n* = 28) and 59.65% (*n* = 68) of isolates were non-biofilm producers, weak, medium, and strong biofilm producers, respectively. The distribution among biofilm-producers among non-MDR and MDR *A. baumannii* isolates is shown in Table 1.; no significant differences were observed between non-MDR vs. MDR isolates regarding their distribution of biofilm-production levels (*P* = 0.655; φ: 0.123).

**Table 1.** Distribution of environmental *A. baumannii* isolates in the context of biofilm-production

	Non-biofilm producer	Weak biofilm-producer	Medium biofilm-producer	Strong biofilm-producer	Overall
Non-MDR isolates	5 (4.39%)	6 (5.36%)	15 (13.16%)	32 (27.97%)	58 (50.88%)
MDR isolates	2 (1.75%)	5 (4.39%)	13 (11.49%)	36 (31.49%)	56 (49.12%)
Overall	7 (6.14%)	11 (9.65%)	28 (24.65%)	68 (59.65%)	114 (100.00%)

### Disinfectant susceptibility in environmental *A. baumannii*

Medium (*n* = 28) and strong (*n* = 68) biofilm-producing *A. baumannii* isolates were subjected to disinfectant susceptibility testing. Table 2 presents the MICs of disinfectants for the four agents tested. The MIC ranges for the compounds were as follows: benzalkonium chloride 16–128 μg mL^−1^, chlorhexidine digluconate 4–128 μg mL^−1^, formaldehyde 64–256 μg mL^−1^ and triclosan 2–16 μg mL^−1^, respectively. The lowest MIC_90_ value was observed for triclosan (4 μg mL^−1^), while the highest was shown in the case of formaldehyde (128 μg mL^−1^).

**Table 2.** Minimum inhibitory concentrations (MICs) of disinfectants against environmental *A. baumannii* isolates

	MICs (μg mL^−1^)									
Disinfectant	2	4	8	16	32	64	128	256	MIC_50_	MIC_90_
Benzalkonium chloride *n* = 96 (100.00%)	–	–	–	*n* = 10 (10.42%)	*n* = 22 (22.92%)	*n* = 55 (57.29%)	*n* = 9 (9.37%)	–	64	64
Chlorhexidine digluconate *n* = 96 (100.00%)	–	*n* = 3 (3.13%)	*n* = 11 (11.46%)	*n* = 18 (18.75%)	*n* = 42 (43.75%)	*n* = 18 (18.75%)	*n* = 4 (4.16%)	–	32	64
Formaldehyde *n* = 96 (100.00%)	–	–	–	–	–	*n* = 23 (23.96%)	*n* = 65 (67.71%)	*n* = 8 (8.33%)	128	128
Triclosan *n* = 96 (100.00%)	*n* = 80 (83.33%)	*n* = 8 (8.33%)	*n* = 5 (5.21%)	*n* = 2 (3.13%)	–	–	–		2	4

## Discussion

*A. baumannii* is an ubiquitous microorganism, which is commonly isolated from nosocomial environments and from the skin of hospitalized individuals [39]; furthermore, it has emerged as one of the pathogens with highest levels of MDR, leading to considerable difficulties in the treatment of these infections [40, 41]. Additionally, during the COVID-19 pandemic, HAIs with MDR *Acinetobacter* spp. were a significant risk factor for worse outcomes in affected patients [42]. The eradication of these pathogens is a serious challenge, due to microbial biofilm-production, conferring protection from noxious environmental factors and antimicrobials (MICs of drugs may increase 100–10,000-fold, due to insufficient penetration into the deep layers of biofilm) [43, 44]. In our current laboratory study, the biofilm-forming capacity, antimicrobial and antiseptic resistance rates of environmental *A. baumannii* were assessed. We have shown that the majority of environmental isolates (84.30%) were moderate or strong biofilm-producers, which was similar to the rates (78.32%) detected from clinical isolates in our previous study [24]. The propensity for biofilm-formation was further highlighted by Zeighami et al., where all isolates (100%) originating from ICUs were either moderate or strong biofilm producers [45]. The meta-analysis of Gedefie et al. – taking into account studies up to 2022 – reported a pooled prevalence of 65.63% for biofilm-formation in clinical *A. baumannii*; additionally, biofilm-forming isolates were classified as “strong”, “mild”, and “weak” producers of biofilm in 41.34%, 33.57% and 27.63% of cases [46].

Antibiotic resistance rates were highest against the tested fluoroquinolones, followed by trimethoprim-sulfamethoxazole, carbapenems and aminoglycosides. In contrast, in the case of clinical isolates, carbapenem-resistance had a lower, while aminoglycoside-resistance had a higher prevalence, respectively [24]. Nonetheless, colistin had largely retained its effectiveness in both isolate groups, which corresponds to other reports in the literature [47]. Phenotypic expression of efflux pumps was substantially higher in environmental isolates (48.25% vs. 27.51%), while MDR rates were similar (49.12% vs. 42.72%), although this could have been influenced by the difference in the number of isolates involved in the two studies [24]. In the experiments of Hassan et al., over 90% of *A. baumannii* isolates were resistant against most antibiotics tested, although the genotyping of all genetic determinants of resistance was not carried out. Moderate and strong biofilm-producers constituted >64% of isolates characterized [48]. The meta-analysis of Salmani et al. – taking into account studies between 2000 and 2019 – reported a combined biofilm-formation rate of 69.1% from clinical *A. baumannii*, in addition to highlighting that the prevalence of MDR in biofilm-forming isolates was 96.1% [49].

The association between the presence and extent of biofilm-production, expression of various resistance genes and the MDR phenotype has been subject to considerable interest [50]. Nevertheless, based on the evidence currently available, it is unclear if their co-occurence is simply due to chance, or whether there is some underlying mechanism present (e.g., adaptational changes to gene expression, fitness costs) [51, 52]. Our results showed no significant differences between biofilm-forming capacity of environmental *A. baumannii* in the context of MDR; these findings corroborate our previous findings on clinical isolates [24]. On the other hand, several studies found significant associations (albeit in varying directions) between the two protective mechanisms. For example, a meta-analysis aiming to collect evidence on clinical *Pseudomonas aeruginosa* isolates by MirzaHosseini et al. – including published articles between 2000 and 2019 – showed higher rates of MDR in strong biofilm-producing isolates, with over >50% of articles in agreement [53]. The study Kasperski et al. also highlighted the high prevalence of strong biofilm-forming *A. baumannii* among XDR isolates, originating from ICU patients [54]. Some authors associate high-levels of biofilm-formation to the presence or absence of specific factors; such are the studies of Azizi et al. [55], which noted that *A. baumannii* carrying the *bla*_PER-1_ beta-lactamase were successful biofilm-producers (compared to non-carriers, as those isolates were less efficient in adhering to epithelial cells), and the study of Gallant et al. [56], noting that *P. aeruginosa* isolates expressing the *bla*_TEM-1_ beta-lactamase were limited biofilm-producers (compared to non-carriers, due to loss of adhesive potential of these strains). Interestingly, the experiments of Zeighami et al. showed that porin-deficient mutants of *A. baumannii* – which showed fenotypic resistance to numerous antibiotics – had significantly lower biofilm-forming capacities, due to deficiencies in bacterial attachment and aggregation [45]. Similarly, Qi et al. showed that susceptible *A. baumannii* isolates were more prolific biofilm-producers, compared to their MDR counterparts [57].

Finally, the antiseptic susceptibility of biofilm-forming *A. baumannii* isolates were assessed against four agents: lowest overall MICs were measured for triclosan, while highest concentrationst were needed from formaldehyde. From the context of effective IPC measures (e.g., hand hygiene, antiseptic showers, cleaning of hospital wards, treatment of medical equipments), the availability of effective antiseptic and disinfectant agents is crucial, especially against microorganisms that may persist in biofilm [58, 59]. The effectiveness of triclosan and high MICs for formaldehyde has been demonstrated in other studies for various pathogens [60, 61]. The study of Kheljan et al. found similar MIC_50_ and MIC_90_ values for the same antiseptics tested in clinical *A. baumannii*. During the genetic characterization of efflux pump genes in the isolates, the presence of the *qacED1* gene affected the MICs of all antiseptics, while detection of the *qacE* and *aceI* genes only affected MIC of chlorhexidine digluconate and benzalkonium chloride [38]. The study of Lanjri et al. compared the antiseptic and disinfectant susceptibilities of clinical and environmental *A. baumannii* isolates using the broth microdilution method. 1:3 dilution of povidone-iodine (4% solution), pure solution of 70% ethyl-alcohol, 1:100 dilution of chlorhexidine digluconate (0.5% solution) and 1:1000 dilution of N-(3-Aminopropyl)-N-dodecylpropane-1,3-diamine (51 mg g^−1^ and 25 mg g^−1^) were effective against all tested isolates; interestingly, environmental isolates were most susceptible to the tested compounds than clinical isolates [17]. Furthermore, the comprehensive study of Saperkin et al. assessed the disinfectant susceptibility rates of over 400 *A. baumannii* isolates from hospital environments, against >40 disinfectants from four different chemical classes. They found highest resistance rates against oxygen-based compounds, while quaternary ammonium compounds and amines remained the most effective [62].

## Conclusions

*A. baumannii* is a serious concern in healthcare-associated infections of immunocompromised patients, due to the increasing prevalence of extensively-resistant isolates. The present study revealed that *A. baumannii* displayed high levels of resistance to commonly used antibiotics in clinical practice, with nearly half of the isolates being MDR. The findings of this study also confirmed our previous observations with clinical isolates, that is, the MDR status of the isolates did not influence their proclivity to biofilm-formation. Additional studies are warranted to ascertain the co-occurrence of antimicrobial resistance and potent biofilm-formation in its full capacity. Furthermore, our study demonstrated the retained effectiveness of several disinfectants *against A. baumannii.* The conscientious use of disinfectants and antiseptics, together with periodic surveillance on susceptibility trends, is essential to curb the spread of these bacteria, and to maintain current infection prevention capabilities in healthcare settings.

## Funding

None.

## Conflicts of interest

The authors declare no conflict of interest, monetary or otherwise. The authors alone are responsible for the content and writing of this article.

## Data availability statement

All data generated during the study are presented in this paper.
